# Public policy, health system, and community actions against illness as platforms for response to NCDs in Tanzania: a narrative review

**DOI:** 10.3402/gha.v7.23439

**Published:** 2014-05-15

**Authors:** Emmy Metta, Beverly Msambichaka, Mary Mwangome, Daniel J. Nyato, Marjolein Dieleman, Hinke Haisma, Paul Klatser, Eveline Geubbels

**Affiliations:** 1 Ifakara Health Institute, Dar es Salaam, Tanzania; 2 Royal Tropical Institute, Amsterdam, The Netherlands; 3 Faculty of Spatial Sciences, Population Research Centre, University of Groningen, Groningen, The Netherlands

**Keywords:** NCD, policy, health system, community response

## Abstract

**Background:**

Most low- and middle- income countries are facing a rise of the burden of non-communicable diseases (NCDs) alongside the persistent burden of infectious diseases. This narrative review aims to provide an inventory of how the existing policy environment, health system, and communities are addressing the NCDs situation in Tanzania and identify gaps for advancing the NCD research and policy agenda.

**Methodology:**

A literature search was performed on PubMed and Google scholar with full text retrieval from HINARI of English language articles published between 2000 and 2012. Documents were read to extract information on what Tanzanian actors were doing that contributed to NCDs prevention, treatment, and control, and a narration was written out of these. Reference lists of all retrieved articles were searched for additional relevant articles. Websites of organizations active in the field of NCDs including the Government of Tanzania and WHO were searched for reports and grey literature.

**Results:**

Lack of a specific and overarching NCD policy has slowed and fragmented the implementation of existing strategies to prevent and control NCDs and their determinants. The health system is not prepared to deal with the rising NCD burden although there are random initiatives to improve this situation. How the community is responding to these emerging conditions is still unknown, and the current health-seeking behavior and perceptions on the risk factors may not favor control of NCDs and their risk factors.

**Conclusion and recommendation:**

There is limited information on the burden and determinants of NCDs to inform the design of an integrative and multisectorial policy. Evidence on effective interventions for NCD services in primary care levels and on community perceptions on NCDs and their care seeking is virtually absent. Research and public health interventions must be anchored in the policy, health system, and community platforms for a holistic response.

Tanzania’s population of 44.9 million in 2012 is more than three times the 12.3 million estimated in 1967 (1). The population growth rate is estimated at 2.7% annually with a crude birth rate of 12.9 per 1,000 inhabitants. The fertility rate at 5.4 children per woman in 2012 is a drop from the 6.5 and 6.3 children per woman estimated in 1988 and 2002 respectively, but is still very high and contributes to the rapid increase in population (1). The Tanzanian demographic profile, like that of other low- and middle-income countries, is largely young, with 42.5% of the population being younger than 15 years, 51.9% between 15 and 59 years, and 5.6% aged 60 years or older (1). The increasing life expectancy at birth from 50 years in 1988 to 55 years in 2010 (2) is, however, changing this picture by slowly increasing the middle-aged and older populations. This improved survival has been attributed to improved health standards and hence reduced mortality (3). Eighty percent of Tanzania’s population lives in rural areas (1), and the adult literacy level was 71% in 2010 (4). Tanzania’s gross national income per capita was USD 551 in 2010 (4), making it one of the poorest countries worldwide. Health sector spending amounted to 7% of the national gross domestic product in 2011 (5), with the government contributing to 53% of all health expenditure in 2010/11 fiscal year (6).

Tanzania has for decades struggled with the burden of infectious and deficiency disorders along with poor maternal and child health indicators. Successes have been documented in the form of a steady decline of the maternal mortality ratio from 529 deaths in 1996 to 452 deaths per 100,000 live births in 2010 (4); and the under-5 mortality ratio from 137 in 1978 to 81 deaths per 1,000 live births in 2010 (4). Malaria attributable child mortality decreased from 53 in 1999 to 36 in 2007–2008 (7, 8), a reduction in HIV prevalence among persons aged 15 to 49 years was seen from 7% in 2003–2004 to 5.1% in 2012 (9, 10), and evidence of the impact of antiretroviral treatment on population mortality has been documented (11). Although these efforts are ongoing amidst resource constraints (2), the rising prevalence of degenerative non-communicable diseases (NCDs) is exerting further strains on the meager resources.

In 2010, NCDs were responsible for 27% of all deaths in Tanzania (12), a figure that is comparable to that of neighboring countries like Mozambique (13). As in the rest of the world, cardiovascular diseases, cancers, diabetes, and chronic obstructive respiratory diseases (CORDs) have been highlighted as main contributors to premature mortality (14). In 2012, the prevalence of hypertension in Tanzanian adults of 25–64 years of age was 26% and that of raised fasting blood glucose in the same population was 9.1% (15). Findings of a study conducted in selected rural and urban communities in Tanzania in 2003 found that crude yearly stroke incidences were 95 per 100,000 and 107.9 per 100,000, respectively (16). Despite this shrinking rural–urban difference in burden, it has been found that the availability of NCD diagnostic and management services is twice as much in urban compared to rural areas (17). National representative data on heart diseases, cancers, and CORDs could not be obtained. These NCDs are reported to share four main risk factors: poor dietary habits, excessive alcohol use, tobacco use, and lack of physical exercise (18).

The health system is anchored on 5,416 health facilities, at an average ratio of 1.5 facilities per 10,000 persons, with dispensaries being the point of first contact for the majority of the population. Two-thirds of facilities are government-owned, and 236 of the facilities are of hospital level and above (19). The provider to population ratio in 2012 was 7.1 per 10,000 population including only professional health workers. Efforts to tackle infectious diseases have been targeting the health system, policies, and the community, which are important constituents of any health sector response. This paper is an inventory of how the policy environment, health system, and communities are addressing the NCDs situation. It highlights the ways to adapt these platforms for responses to the increasing burden of NCDs and identifies the knowledge gaps for advancing the research and policy agendas for NCDs in Tanzania.

## Methodology

**Fig. 1:**
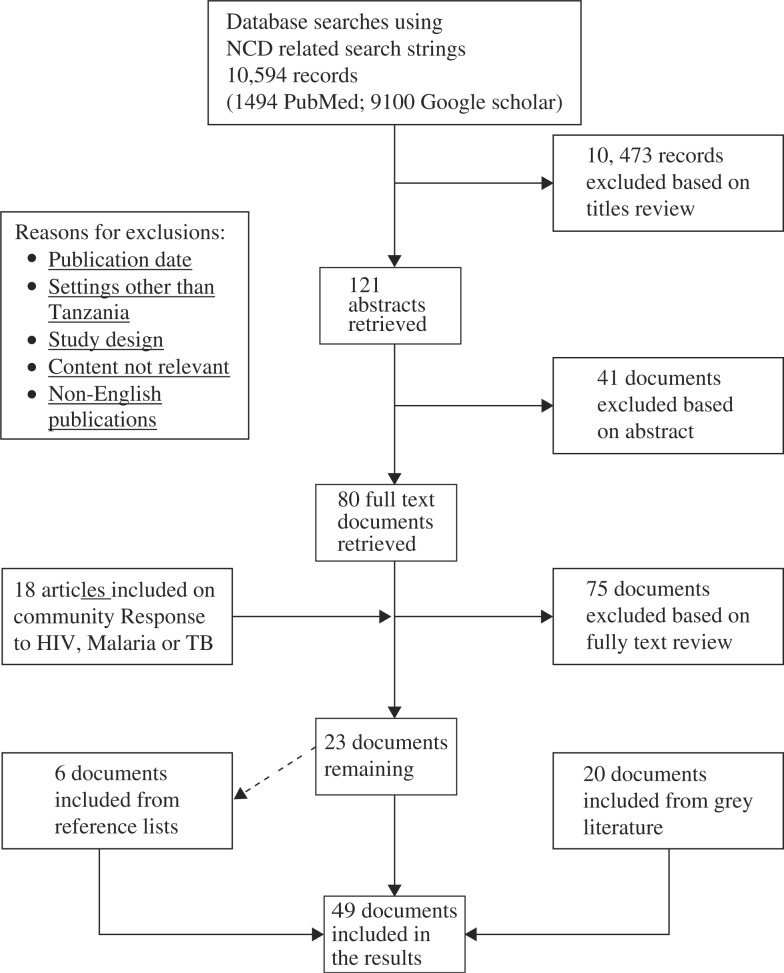
The process of selecting articles.

A literature search was performed using a set of comprehensive topic-related search terms. Inclusion criteria were English-written articles on original work conducted in Tanzania. We excluded systematic or narrative reviews, opinion papers, documents including expectant women as participants, and articles on drug evaluations and diagnostics. The search was restricted to publications between 1 January 2000 and 31 December 2012 to ensure that the retrieved articles reflected the current situation and most recent responses. The search was performed on PubMed and Google scholar, with full text articles retrieved from HINARI. We also undertook targeted grey literature search, focusing on large institutions including WHO, NCD interest groups in Tanzania, and documents and reports of the government of Tanzania. Articles selected included primary research articles, evaluation, and situation analyses reports. The references of retrieved articles were manually searched for additional material. The search terms were based on key terms aligned to policy, health care system, and community, the important constituents of a health sector response (20). The terms were combined by Boolean operators ‘AND’ to narrow the search appropriately and ‘OR’ to expand it with similar terms. The search strategy included the following strings: ‘non-communicable diseases’ OR ‘non communicable diseases’ OR ‘NCDs’ AND Tanzania; ‘non-communicable diseases’ OR ‘non communicable diseases’ OR ‘NCDs’ AND health services OR health care AND Tanzania; ‘non-communicable diseases’ OR ‘non communicable diseases’ OR ‘NCDs’ AND health seeking OR health-seeking AND Tanzania; ‘non-communicable diseases’ OR ‘non communicable diseases’ OR ‘NCDs’ AND policy AND Tanzania. The search resulted in 10,594 articles as depicted in Fig. 1. Ten thousand, four hundred and seventy-three of these were excluded for reasons including title not focused on NCDs, language other than English, or publication date was out of target. One hundred and twenty-one titles were found to be relevant to our subject area and their abstracts were retrieved and screened to determine if they matched our criteria. Forty-one abstracts were excluded based on content relevance to our topic and study design. From this step, 80 abstracts were identified to be relevant and their full text versions were retrieved from HINARI. Bibliographies of retrieved documents were also searched for relevant papers. Six documents were identified this way, three of which were peer-reviewed articles, and the other three were reports. When our initial search yielded only two papers on community responses to NCD, we expanded our inclusion criteria to include articles on health seeking for HIV, TB, or malaria. We obtained 18 relevant publications based on this expansion.

Analysis was done based on pre-determined themes on NCD policy prevention and control, health system financing, NCD health services, and human recourses of health regarding NCD services and community health-seeking behavior and practices. These themes were pre-determined taking into account the review objectives and focused on the main health sector response constituents. Documents were handled manually in that no data management software was used during the analysis. They were read to extract information on what Tanzanian actors were doing that contributed to NCDs prevention, treatment, and control, and a narration was written out of these responses. There were discussions among the first four authors on content of the results and in case of disagreements resolution was by consensus.

## Results

This review included 49 documents that were comprised of 26 peer-reviewed articles which were mainly cross-sectional studies published between 2006 and 2012. Three of the 26 articles are included in the policy section, one in the health systems section, and 22 in the community section. The other 23 documents were grey literature mainly from the Tanzanian government and WHO sources, published between 2001 and 2012. Six of these documents were reviewed for the policy section, 17 for the health system, and none was reviewed for the community section. Three of the six documents obtained through searching the reference lists of retrieved articles were peer-reviewed articles, the remaining were grey literature. In these results the NCD-related policy environment is described first and then the preparedness of the health system for the rising NCD burden. This will be followed by an inventory of community responses to NCDs.

### Policies and strategies on NCDs and their determinants in Tanzania

The government has established a unit at the Ministry of Health and Social Welfare (MoHSW) to steer formulation of NCD policies and guidelines (21). It has also included prevention and control of NCDs as strategic objectives of the health sector (7). This National Strategy for NCDs 2008–2013 identifies primary, secondary, and tertiary prevention as important components in addressing NCDs (7). The strategy is integrated and generic and addresses NCDs only from the health sector perspective (7, 12). There was neither an overarching national NCD policy nor evidence of harnessing of non-health sectorial policies in the current response to NCDs despite the multifaceted etiologies of these diseases. Regarding addressing of the four main risk factors of NCDs, by 2011, Tanzania had integrated strategies for alcohol; smoking; physical inactivity; and unhealthy diets, overweight, and obesity (12).

The government signed (in 2004) and approved for implementation (in 2007) the WHO’s Framework Convention for Tobacco Control (FCTC) (22). This convention clearly presents both demand and supply reduction provisions for controlling exposure to tobacco, among other provisions (23). Fortunately, some of its demand–reduction provisions such as the introduction of excise taxation on tobacco were already in place (24), but still more actions are needed considering that smoking tobacco costs the nation more than 30 million dollars annually in treatment of tobacco-related cancers (25). In 2011, it was reported by WHO that Tanzania had implemented none of the five tobacco (m)POWER measures to the highest level of achievement (12). Concerning control of the use of alcohol, Tanzania’s alcohol policy includes excise taxes on alcohol, a minimum drinking age of 18, a zero tolerance policy for drinking and driving, regulations on alcohol advertising and sponsorships, and restrictions for on- and off-premise sales of alcoholic beverages (26).

The scope of the current Tanzania National Nutrition Strategy states: *the strategy seeks to ensure the nutritional status of all citizens of Tanzania throughout their life cycle* (27). However, the strategy focuses more on women of reproductive age and children under 5 years of age with special emphasize on children less than 2 years of age because malnutrition’s most serious and lasting damage occurs during pregnancy and the first two years of life. None of the targets set are related to NCDs or their dietary risk factors.

There was no policy on physical exercise that was found other than a reference to a policy on physical education in schools (28).

### Health systems and the response to NCDs in Tanzania

In Tanzania, health services for NCDs are mostly provided from district hospitals to higher level health facilities (29). In 2010, the public health system had only two out of eight NCD-related screening and diagnostic tests nationwide (cervical cancer screening and breast cancer screening) at primary level facilities (21).

In regards to medications, by 2009, the problem of stock-outs for all drugs including for NCDs was persistent and was associated with poor health outcomes, especially for those who could not access services in the private facilities (30). In 2010, 15 out of 17 NCD-related drugs were available in public health system (21).

The Human Resource for Health (HRH) deficit in Tanzania stood at 65% in 2008 (7, 19). With the projected increase of the NCD burden, the government is re-thinking its HRH management strategies (7, 19). The immediate strategy involves capacity building of HRH for NCD care in the form of in-service trainings offered by various disease-interest groups (29, 31) such as the Tanzania Diabetes Association which has trained staff from even lower level facilities in the Lake Region (Zachariah Ngoma, personal communication, 26 March 2013). The introduction of NCD prevention and control modules into courses in local training institutions (32) is availing the training to a wider pre-service trainee population, which may help to build the HRH pool of different cadres for the future. The Association of Private Health Facilities in Tanzania (APHFTA) has conducted in-service trainings for diabetes and hypertension care in 18 out of 27 regions of Tanzania (33). In the public health system, comprehensive clinical guidelines have been developed only for diabetes and hypertension (21) but evidence for their distribution, training, and utilization is limited.

Currently, both health facility and population-based information systems reflect limited measurement of NCD-related variables (34, 35). The standards of Health Management Information System (HMIS) in Tanzania are slowly improving with efforts underway to minimize the existing challenges in quality and utilization of health information and to expand information sources such as the cancer registry (36). For population-based NCD information to inform policy and other interventions, the MoHSW resorted to search for evidence on NCDs and their risk factors through projects. The National Institute for Medical Research conducted WHO STEP wise approach to chronic disease risk factor surveillance (WHO STEPs survey), to quantify the risk factors and burden for NCDs (15) nationally. Variables for NCD risk factors have been included in the Tanzania demographic health surveys (DHS) tool (34) and into the MZIMA adult health community cohort (37). There is lack of literature on utilization of even wider existing platforms such as the sentinel Panel of Districts (SPD) (38).

Underfunding of health services is a persistent challenge contributing to a myriad of other malfunctions in the system (39). As a signatory of the Abuja declaration 2001 which required Governments to allocate at least 15% of its annual budget to health sector, Tanzania allocated 8.9% for the 2011–2012 fiscal year (40). Financing of health care is through, among others, health insurance mechanisms including community health funds that are being rolled out, though uptake is reported to be low (41). Integration of services is one way to deal with NCDs proposed in the Health Sector Strategic Plan III and provides a cost-efficient means of quality service delivery (7). The government has collaborated with other stakeholders in a private hospital in Dar es Salaam whereby NCD services were introduced in the HIV clinic and staff trained in management of co-morbidities. In 2011, this initiative was evaluated and results showed that 15% of its 3,400 patients on antiretroviral therapies had co-morbidities including hypertension, hyperlipidemia, diabetes, and other metabolic disorders (42). Other NCD-related programs that attest to advantages of private–public partnership include the Ocean Road Cancer Institute, and the APHFTA (33, 43).

### Communities in preventive health and health care seeking

In public health, community involvement is key to achieving the goals of preventive and promotional health programs (44). In Tanzania the importance of involving communities in effective disease control interventions for various infectious diseases has been well documented (45). This practice was associated with increased access and acceptance of palliative services (46), effectiveness and efficiency of disease control interventions (47), as well as equity, sustainability, and communities’ self-reliance (48). Private facility initiatives such as those under APHFTA through their NCD program, involved primary schools and communities around health facilities reached by their program in healthy lifestyle teachings including healthy eating and physical activities (33). The impact of these interventions however, is not yet known.

Factors contributing to development of NCDs include social determinants which are highly linked to complex sociocultural practices and beliefs, making it challenging to effect lifestyle changes (49). For example, fatness was associated with beauty and economic prosperity of the household (50); and excessive alcohol intake was fuelled by cheaply available local brew, and the need for entertainment and relaxation on the part of users (51).

Literature suggests that where illness symptoms were thought to have spiritual etiology, traditional healers became the preferred source of treatment (52, 53–54), because they were perceived to have ‘appropriate skills’ for managing diseases considered as *out of order* (55).

Socioeconomic determinants have been shown to influence the timeliness of peoples’ responses to the utilization of emergency and in-patient services (56) and the initiation and continuation of treatments for chronic illnesses (55), all of which influence patient outcomes (57). Studies revealed that services at health care facilities are only sought when chronic illness symptoms persist or worsen, after using over-the-counter medicines (58, 59). In addition, accessibility to health facilities has been shown to facilitate prompt care seeking (60) and continuity of care for chronic illnesses (61).

Several factors influence adherence to treatments to infectious disease in Tanzania including; people’s perceptions of the illness and quality of medicines (62), fear of side effects (63), patient–provider relationships, cultural pressure, cost and availability of medicines (61), and the patient’s understanding of the medication schedule (64). Others include poor access of re-filling prescriptions, inadequate nutrition especially for medicines perceived to increase hunger (63), and long hours of absence from home (65). Adherence to medications was reported to also be influenced by gender (65).

## Discussion

Our findings have shown that there is no existing NCD policy in Tanzania. For clarity of vision and purpose and coherence of interventions, a national policy is important (66, 67). Reasons for lack of a policy include the lack of evidence to inform such a policy (67). There are several opportunities in available guidelines and strategies for controlling exposure to the major NCD risk factors, implementation of which is partly hampered by lack of an NCD policy. Being multifaceted in their etiology, NCD policies being designed must as well be multisectorial. In Tanzania, there was no evidence of including other sectors such as education and agriculture in policy development for NCD prevention and control. The country could learn from experiences shaping the HIV response where multisectoral approaches have been adopted in prevention and communication activities (68). Regarding prevention of exposure to NCD risk factors, the adoption and implementation of FCTC is a positive step towards tobacco control. However, more effort is needed towards fully implementing the convention through the mPOWER measures.

Although some aspects of alcohol policy are in place, implementation of these, especially in rural areas where local brew is mostly produced and consumed, will be a challenge (68). Addressing diet-related NCDs in the national nutrition strategy without setting targets related to NCDs or their dietary risk factors indicates that overnutrition is not yet prioritized. Whereas there are still no policies focusing on physical activity, it has been proposed that the policies will have to address activity at work places, leisure activities, and means of transport (69). Physical activity policies should also be sensitive to sociocultural differences across Tanzania.

As policies are being developed to tackle the rising NCD burden, the health system in Tanzania must gear up for roles in primary, secondary, and tertiary prevention of NCDs. Concentration of the NCD services at higher level health facilities leads to late diagnosis and delayed access to appropriate treatments, especially in rural areas (70). Accessibility of NCD services also entails availability of diagnostic and treatment services at the primary level facilities. Lack of screening and diagnostic tests at primary level facilities creates a challenge for NCD care, especially the long-term patient monitoring and management. Whereas the WHO observatory indicates that NCD medication availability was impressive, it is the consistent medical supply of affordable medicines that contributes to favorable health outcomes. A recent national survey in Tanzania showed that, only 9% of rural and 20% of the urban health facilities provided diabetes diagnosis and management services and only half of these had the staff, drugs, diagnostics, and guidelines to actually provide the service on the day of the survey (17). Generally the health care system is not prepared to tackle the rising burden of NCDs (17, 71). The HRH crisis hinders quality preventive, diagnostic, and treatment service provision for NCDs close to the community and requires creative thinking on how to more efficiently use the existing HRH. The pre-service and in-service training efforts, though prudent, do not completely address the shortage of competent cadres in lower level facilities in the country. Creativity including task shifting where lower cadres of HRH are prepared for NCD prevention and management could ease this crisis as has been done elsewhere (72). Availing tools such as clinical guidelines has facilitated task shifting and could be adopted in Tanzania’s primary care facilities (72).

The rising burden of NCDs will increase the financial strain on the health system because of their chronic nature; hence, new approaches to minimize costs of interventions must be embraced. Integration ensures that, ‘clients receive a continuum of preventive and curative services, according to their needs over time and across different levels of the health system’ (73).Various integration models are possible, but little has been documented on pilot projects. The benefits of integration of services that have been observed in the Tanzanian example have been demonstrated in other settings where chronic diseases clinics have been piloted (74). This idea of chronic disease clinics could be replicated in primary care settings where HIV prevention, care, and treatment services are offered.

Improvement of HMIS would positively influence quality of care for NCD patients and overall health system functioning (75). Systematic information concerning public awareness and practices regarding NCD risky behaviors as well as factors that shape risky lifestyle is still limited. Existing population survey platforms such as the demographic surveillance system and SPD could be adapted to fit chronic disease surveillance by introducing outcome measures that realistically measure morbidity burden of NCDs. Mortality outcomes may not capture true burden of NCDs whose onset and course is insidious.

The financial strains caused by NCDs are also experienced by patients and their families. Ensuring effective implementation of the policy on health care fee waivers which exempts patients with chronic diseases from user fee charges will improve accessibility and equity in utilization of NCD services. However, this has implications to the government resources and may necessitate government’s re-allocation of its financial resources to the health sector.

Considering that NCDs are insidious in their onset, slow in their progression, and long-term, communities have to be involved in their prevention and control (76). Community involvement has been shown to be effective in other chronic diseases prevention and control efforts (77, 78). This involvement has also been recommended in the Tanzania HSSP III as an objective in the NCD control strategy (7). Since community responses, including lifestyle changes, are shaped by sociocultural aspects and health system factors, effective mitigation of NCDs will therefore have to integrate community-based and individually targeted interventions (79) that are sensitive to variations in gender and cultural norms, for them to be acceptable (80).

Knowing that health services are commonly sought when illnesses persist or worsen after failure of self-medication, interventions to promote prompt health care seeking must be designed. Self-medication may present a challenge when it comes to NCDs, most of which are initially asymptomatic and symptoms indicate worsening of disease intensity. Information is lacking on NCD self-treatment patterns and their motivations, which may hinder the possibility of addressing any undesirable patterns in the community.

As NCD medication use is long-term, it is important to understand how sociocultural aspects shape people’s sustained compliance to medicines. Compliance to long-term medication has been studied for infectious chronic diseases (81, 82); however, community perceptions towards infectious disease and NCDs may not be the same. Therefore application of the findings from these studies to NCDs medication may not be valid. Exploration of the aspects that might shape sustained compliance to long-term NCDs medication would inform designing of strategies that foster compliance to long-term treatments.

The knowledge gaps for both research and policy agendas regarding responses to the rising NCDs burden in Tanzania include inadequate information on the epidemiological patterns of NCDs and their determinants across Tanzania and the limited awareness of the local context to inform the design of an integrative and multisectorial policy. Apart from better understanding the epidemiological pattern of NCDs, it will be important to project the implications of the current dual burden on population life expectancy, taking into account not just the health systems and policy environment, but also the wider demographic and development indicators. There is limited evidence on effective interventions for NCD services in primary care levels and information on community perceptions on NCDs and their care seeking is virtually absent.

### Limitations and strength of the review

This review included mainly published documents and thus *omitted* insights from non-published developments. Through the inclusion of only English language documents we might have missed relevant results published in Kiswahili. However, a Kiswahili language research journal does not exist in Tanzania and English is one of the country’s official languages. We therefore think that this has not resulted in non-inclusion of formal government- produced documents nor of research publications. The search terms did not include ‘chronic disease’ because not all chronic diseases are NCDs. We thus may have missed articles that used only ‘chronic disease’ as a term to describe NCDs. Nevertheless, the holistic nature of the information reviewed provides a broader view of the NCDs situation and the responses in Tanzania. The narrative review approach was chosen rather than a systematic review because it is well suited to present a broad perspective on a newly emerging problem (83, 84).

## Conclusions and recommendations

NCDs and their risk factors are largely lifestyle related, making multisectorial responses unavoidable. These conditions are posing a critical challenge to the government, health system, and communities that have to face both communicable and NCDs. There is limited context-relevant information on the burden and determinants of NCDs which may hamper the design of effective interventions, especially for prevention. The existing research platforms such as the health and demographic surveillance system (HDSS), DHS, and SPD can be leveraged to address these NCD knowledge gaps.

Current efforts to address NCDs in Tanzania are fragmented due to lack of a NCD policy. The success of NCD prevention and control requires such a clearly defined NCD policy to provide a roadmap for implementation of multisectoral strategies and plans. For effective interventions targeting NCDs risk factors at local level, empowerment of local government authorities tasked with implementation of existing policies in the country is needed.

The country’s health system is not adequately prepared to accommodate the requirements of NCDs. There is an urgent need to design and evaluate low-tech, low-cost interventions for prevention, diagnosis, treatment, and continuity of care that can be scaled up at primary care levels. This may necessitate the adoption of an integrated health care model to address both NCDs and other chronic communicable diseases as a strategy to address the HRH and financing challenges. Also, supporting informed decision making for NCDs at clinical and policy levels requires accurate clinical record keeping and diseases registry maintenance for NCDs and their risk factors.

Information on community awareness and practices in prevention and health care seeking regarding NCDs is limited. The role of the community in NCD development, prevention, and management must be explored by understanding the motivations for health-seeking, self-medication practices, and aspects shaping continuity of care in their particular contexts.

**Main findings**

- There is limited context-relevant information on the epidemiological patterns of NCDs and their determinants across Tanzania, which may hamper the design of effective interventions, especially for prevention.
- Current efforts to address NCDs in Tanzania are fragmented due to lack of a NCD policy.
- The county’s health system is not adequately prepared to accommodate the requirements of NCDs, and the information on community awareness and practices in prevention and health care seeking regarding NCDs is limited.

**Key messages for action**

- The existing research platforms such as HDSS, DHS, and SPD should be leveraged to address the NCD knowledge gaps in the epidemiological patterns, in best practices in health care, and in community roles in prevention and management of NCDs in Tanzania.
- Considering the multifaceted nature of NCD risk factors and causation, there is a need to formulate a clear, multidimensional policy on NCDs prevention and management.
- There is an urgent need to design and evaluate low-tech, low-cost interventions for prevention, diagnosis, treatment, and continuity of care that can be scaled up at primary care levels. This may include the adoption of an integrated health care model to address both NCDs and other chronic communicable diseases as a strategy to address the HRH and financing challenges.
